# Genetic Association and Expression Studies Indicate a Role of *Toll-Like Receptor 8* in Pulmonary Tuberculosis

**DOI:** 10.1371/journal.pgen.1000218

**Published:** 2008-10-10

**Authors:** Sonia Davila, Martin L. Hibberd, Ranjeeta Hari Dass, Hazel E. E. Wong, Edhyana Sahiratmadja, Carine Bonnard, Bachti Alisjahbana, Jeffrey S. Szeszko, Yanina Balabanova, Francis Drobniewski, Reinout van Crevel, Esther van de Vosse, Sergey Nejentsev, Tom H. M. Ottenhoff, Mark Seielstad

**Affiliations:** 1 Infectious Diseases, Genome Institute of Singapore, Singapore, Singapore; 2 Department of Immunohematology and Blood Transfusion, Leiden University Medical Center, Leiden, The Netherlands; 3 Eijkman Institute for Molecular Biology, Jakarta, Indonesia; 4 Human Genetics, Genome Institute of Singapore, Singapore, Singapore; 5 Department of Internal Medicine, Padjadjaran University, Hasan Sadikin Hospital, Bandung, Indonesia; 6 Juvenile Diabetes Research Foundation/Wellcome Trust Diabetes and Inflammation Laboratory, Cambridge Institute for Medical Research, Department of Medical Genetics, University of Cambridge, Cambridge, United Kingdom; 7 Health Protection Agency Mycobacterium Reference Unit and Clinical TB and HIV Group, Center for Infectious Diseases, Institute for Cell and Molecular Sciences, Barts and the London Medical School, London, United Kingdom; 8 Samara Region Tuberculosis Service, Samara, Russian Federation; 9 Department of Internal Medicine, Radboud University Nijmegen Medical Center (RUNMC), Nijmegen, The Netherlands; 10 Department of Infectious Diseases, Leiden University Medical Center, Leiden, The Netherlands; 11 Department of Medicine, University of Cambridge, Cambridge, United Kingdom; National Institute of Genetics, Japan

## Abstract

Despite high rates of exposure, only 5–10% of people infected with *Mycobacterium tuberculosis* will develop active tuberculosis (TB) disease, suggesting a significant role for genetic variation in the human immune response to this infection. Here, we studied TB association and expression of 18 genes involved in the Toll-like receptor (TLR) pathways. Initially, we genotyped 149 sequence polymorphisms in 375 pulmonary TB patients and 387 controls from Indonesia. We found that four polymorphisms in the *TLR8* gene on chromosome X showed evidence of association with TB susceptibility in males, including a non-synonymous polymorphism rs3764880 (Met1Val; *P* = 0.007, odds ratio (OR) = 1.8, 95% c.i. = 1.2–2.7). We genotyped these four *TLR8* polymorphisms in an independent collection of 1,837 pulmonary TB patients and 1,779 controls from Russia and again found evidence of association in males (for rs3764880 *P* = 0.03, OR = 1.2, 95% c.i. = 1.02–1.48). Combined evidence for association is *P* = 1.2×10^−3^–6×10^−4^. In addition, a quantitative PCR analysis indicated that *TLR8* transcript levels are significantly up-regulated in patients during the acute phase of disease (P = 9.36×10^−5^), relative to baseline levels following successful chemotherapy. A marked increase in TLR8 protein expression was also observed directly in differentiated macrophages upon infection with *M. bovis* bacille Calmette-Guérin (BCG). Taken together, our results provide evidence, for the first time, of a role for the *TLR8* gene in susceptibility to pulmonary TB across different populations.

## Author Summary

One third of the world population is infected with *Mycobacterium tuberculosis*, the bacterium that causes tuberculosis; however, only 5–10% of those infected will develop active disease. Difference in polymorphisms within genes involved in host immune response has been proposed as a plausible reason to explain this phenomenon. Here, we show genetic association of four polymorphisms of *TLR8*, a member of a well-known receptor family involved in pathogen recognition, in an Indonesian population. The association was replicated in males of a follow up cohort from Russia. Expression levels of *TLR8* transcripts and protein showed a marked increase during bacterial infection, confirming our initial findings. To our knowledge, this is the first time that *TLR8* has been associated with response to *M. tuberculosis*. Our results suggest that it may play a significant role in tuberculosis susceptibility and disease activity, and thus should be the focus of concerted studies in human systems.

## Introduction

Although one-third of the world's population is infected with *M. tuberculosis* [1], fewer than 10% of infected –otherwise immunocompetent- individuals will develop clinical disease during their lifetime [2]. The immunological mechanisms that distinguish the majority of individuals who successfully contain these organisms from the minority who develop progressive mycobacterial disease are largely unknown.

It is becoming increasingly clear that innate immunity plays a crucial role in directing many aspects of the host response, including the ensuing adaptive response, making it a primary host defense mechanism. The initial phase of this process is pathogen sensing involving a wide range of pattern recognition molecules. We and others have postulated that pathogen recognition could be a key component in determining the outcome of infection [3],[4]. At the same time, evidence is building in a number of diseases, including TB [5] and meningococcal disease [6], that variations in genes of a related pathway may have similar functional consequences, and thus result in a similar phenotype upon infection.

These observations led us to investigate genetic variants in the Toll-like receptors (TLRs) [7]–[11] and related adaptors for association with human susceptibility to pulmonary TB. So far fifteen functional TLRs have been identified in mammals and implicated in specific recognition of pathogen associated molecules [12]. Upon ligand binding, TLRs initiate a cascade of events leading to the transcription of NFkB-dependent genes, mostly inflammatory genes. All functional TLRs, except *TLR5* (GeneID:7100), were studied. The latter was excluded due to a low level of polymorphism and to complex sequence duplications that could make SNP genotyping difficult. We also studied cytoplasmic TLR adaptors including *MYD88* (GeneID:4615), *TOLLIP* (GeneID:54472), *TIRAP* (GeneID:114609), *TICAM1* (GeneID:148022), *TICAM2* (GeneID:353376) and the downstream signaling molecules, *IRAK1* (GeneID:3654) and *IRAK4* (GeneID:51135), *LY96* (*MD2*) (GeneID:23643) [13] as well as *CD14* (GeneID:929) [14], a surface molecule that partners with *TLR4* (GeneID:7099).

Here we identified four single nucleotide polymorphisms within the *TLR8* gene (GeneID:51311) on chromosome X that confer susceptibility to pulmonary TB in males in an Indonesian population and in a large independent sample of TB patients and controls from Russia. Additional evidence in support of TLR8 (NP_619542.1) in immunity to TB disease came from real-time PCR quantification of elevated levels of TLR8 transcripts (NM_016610.2; NM_138636.3) during active disease, relative to the same individuals following successful completion of anti-TB chemotherapy. In line with this, analysis of differentiated macrophages upon stimulation with BCG over time showed a significant increase of TLR8 expression. Taken together, these results provide strong evidence for the first time, of a role for TLR8 in adult pulmonary TB infection.

## Results

### Genetic Association Analysis

**Figure 1:**
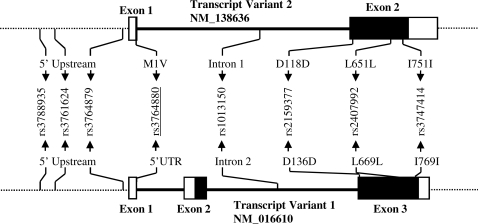
Transcript variants of *TLR8* and location of genotyped SNPs within both transcripts. Exons are shown as rectangles, filled areas represent translated sequence, open areas indicate untranslated regions. The associated polymorphism resulting in a coding change exclusive of transcript variant 2 (rs3764880) is underlined.

**Figure 2:**
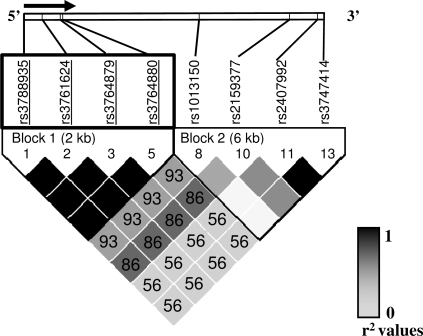
Linkage Disequilibrium Plot and Haplotype Structure of *TLR8*. D' values displayed within each diamond, missing value indicates D' = 100%. Color scheme gradient indicates r^2^ values. At the top, direction of transcription is designated by an arrow. Length of each block, in kilobases (kb), is shown between brackets. Underlined polymorphisms indicated associated SNPs in allelic analysis. Block with significant p-values is displayed within an open rectangle.

Of the 149 SNPs passing quality control as described in materials and methods, allelic and genotypic association analysis identified four SNPs in the *TLR8* gene with nominal p-values below 0.05 (Table 1 and Table S1). We also observed two rare variants, within *TOLLIP* and *TLR9* (GeneID:54106), with significant p-values that were not followed up in this study due to their very low allele frequencies. Three of the associated *TLR8* variants, rs3764879, rs3788935 and rs3761624 localize in the putative regulatory regions, within five kilobases upstream of the gene (Figure 1). The fourth associated polymorphism was a missense variant, rs3764880 (Met1Val), which would ablate the putative start codon in one of the transcripts encoded by this gene. Given that *TLR8* is located on the X chromosome, we performed separate tests for males and females (Table 2). We found a strong allelic association with the minor allele A of the putatively functional polymorphism, rs3764880, with susceptibility to pulmonary TB in males [OR (95% c.i.) = 1.8 (1.2–2.7), P = 0.007]. Very similar and significant association values were found in the three promoter variants, attributable to perfect linkage disequilibrium (r^2^ = 1) between all four polymorphisms (Figure 2).

**Table 1:** Allelic Distribution and Description of SNPs within *TLR8* with p-values<0.05 in Indonesian TB Patients and Controls.

dbSNP rs#	Alleles	No. of Cases^a^	MAF Cases	No. of Controls^a^	MAF Controls	Location^b^	p-value	Permutation p-value^c^	OR (95% c.i.)
rs3764879	G/C	153	0.30	124	0.23	Upstream	0.01	0.038	1.4 (1.06–1.84)
rs3788935	G/A	152	0.30	125	0.23	Upstream	0.014	0.05	1.4 (1.07–1.86)
rs3761624	G/A	152	0.30	126	0.24	Upstream	0.016	0.059	1.4 (1.06–1.8)
rs3764880	G/A	152	0.30	126	0.24	M1V, 5′UTR	0.016	0.059	1.4 (1.06–1.8)

a Number of chromosomes carrying the minor allele.

b Locations for both transcripts encoded by *TLR8* are shown.

c Number of permutations = 10,000.

**Table 2:** Allele Distribution of *TLR8* Polymorphisms among Indonesian TB Patients and Controls by gender.

dbSNP ID	Males					Females			
	No. of Cases (%)^a^	No. of Controls (%)^a^	p-value	Permutational p-value^b^	O.R. (95% c.i.)	No. of Cases (%)^a^	No. of Controls (%)^a^	p-value	O.R. (95% c.i.)
rs3764879	77 (34.6)	49 (21.7)	0.0024	0.012	1.9 (1.2–2.9)	76 (27.1)	74 (24.3)	0.44	1.1 (0.8–1.7)
rs3788935	76 (34.3)	50 (22.1)	0.0039	0.017	1.8 (1.2–2.8)	76 (27.1)	74 (24.3)	0.44	1.1 (0.8–1.7)
rs3761624	76 (34.3)	51 (22.4)	0.007	0.02	1.8 (1.2–2.7)	76 (27.1)	74 (24.3)	0.44	1.1 (0.8–1.7)
rs3764880	76 (34.3)	51 (22.4)	0.007	0.02	1.8 (1.2–2.7)	76 (27.1)	74 (24.3)	0.44	1.1 (0.8–1.7)

a Number and percent of chromosomes carrying the minor allele.

b Number of permutations = 10,000.

In order to address the significance of our findings, a permutation analysis of the allelic p-values was carried out (Table 1). One of the polymorphisms passed the permutation test (N = 10,000), with its p-value remaining statistically significant at an adjusted P<0.05. The same analysis was applied separately by gender. In this case, all four SNPs maintained statistical significance at an adjusted P<0.05 in males (Table 2).

Analysis of genotypes for polymorphisms located on Chromosome X was done using a likelihood ratio test. The same four variants on *TLR8* were found to be more frequent in cases than controls, indicating susceptibility to disease for carriers of the minor allele. Due to the fact that males carry only one copy of each allele, the genotype association outcome was expected to be the same as for the previous allele association result. Thus, we analyzed genotypes of female subjects (Table S2). The observed number of homozygotes for the associated missense polymorphism, rs3764880 (AA), may have been too low to detect an effect (14 affected vs. 9 controls). Nevertheless there was an apparent trend towards the same outcome observed in the overall sample, with affected females showing an increase of homozygotes for the minor allele, compared to the control group.

Investigation of the gene structure of *TLR8* showed two distinct haplotype blocks (Figure 2) in our population. As expected from our initial results, all four associated polymorphisms appeared in the same haplotype block (Block1). Performing separate association analysis of haplotypes in males and females confirmed the genetic association in males (Table S3). The minor haplotype (H2) harboring allele A of rs3764880 showed a pronounced risk effect of disease among male carriers [OR (95% c.i.) = 1.8 (1.2–2.7)]. The population attributable risk of the associated haplotype in males was 4% [15].

In order to confirm these results, genotyping of the four associated polymorphisms was carried out in a follow-up cohort from Russia (1,873 tuberculosis cases, 1,779 controls). Importantly, the minor allele frequency of rs3764880 in the Indonesian population was strikingly different compared to frequencies in the Russian cohort, which was concordant with results obtained for the populations of European and Asian origin from the HapMap project [16]. Around 30% of the Indonesian subjects carried the A allele (Met) associated with risk to TB, whereas this same allele was present in 78% of Russians. Despite these obvious differences in frequencies, the genetic association with pulmonary TB was replicated in the Russian males for allele A of rs3764880 OR (95% c.i.) = 1.2 (1.02–1.48) P = 0.03 (Table 3). Combined evidence for association in males from both Indonesian and Russian populations was P = 1.2×10^−3^–6×10^−4^. Analysis of haplotype blocks showed the same trend of association as observed in the Indonesian cohort. In this case, however, the most common haplotype was the one harboring rs3764880A, and displayed a risk effect among male carriers [OR (95% c.i.) = 1.22 (1.01–1.47)] (Table S3).

**Table 3:** p-value of *TLR8* Polymorphisms in Russian Males and combined (Russian and Indonesian) cohorts.

dbSNP ID	Alleles	Russian cohort				Combined p-value
		No. of Cases (%)^a^	No. of Controls (%)^a^	p-value	OR (95% c.i.)	
rs3764879	G/**C**	1067 (79.7)	994 (76.3)	0.03	1.2 (1.02–1.48)	6×10^−4^
rs3788935	G/**A**	1069 (79.8)	997 (76.4)	0.03	1.2 (1.02–1.48)	9×10^−4^
rs3761624	G/**A**	1070 (79.8)	1000 (76.5)	0.04	1.2 (1.01–1.46)	1.5×10^−3^
rs3764880	G/**A**	1069 (79.7)	997 (76.3)	0.03	1.2 (1.02–1.48)	1.2×10^−3^

a Number and percent of chromosomes carrying the risk allele shown in bold.

### mRNA Expression Study

A subset of 23 patients with active pulmonary TB was selected from the cohort recruited from the outpatient clinic in Jakarta, Indonesia. After informed consent, blood samples were taken from each of the patients during their initial diagnosis with active pulmonary TB disease (at the time of admission), and again following resolution of disease (6 months after completion of a standard anti-tuberculosis multi-drug chemotherapy). Real-time reverse transcription PCR was used to study the mRNA levels of 18 genes (Table S4). We observed that both TLR8 transcripts, TLR8v1 (p = 9.36×10^−5^), TLR8v2 (p = 5.29×10^−5^) and MYD88 (NM_002468.3) (p = 4.09×10^−5^) were the most significantly upregulated in TB patients during active disease, relative to their convalescence. Both variants of TLR8 showed a greater than two-fold increase in expression, whereas MYD88 increased by 1.9-fold (Table 4). Transcript levels of TLR7 (NM_016562.3) and CD14 (NM_001040021.1) were also significantly increased (p = 2.5×10^−3^; 3×10^−2^ respectively), whereas TIRAP (NM_148910.2) showed a downregulation of mRNA expression, fold change = 0.2, but without statistical significance (Table 4).

**Table 4:** Genes tested on mRNA Expression in Acute vs. Convalescence Indonesian TB Samples.

Gene Name	mRNA ID	Fold change	p value	Bonferroni p-value
*TLR1*	NM_003263.3	1.556	5.5*10^−2^	NS
*TLR2*	NM_003264.3	1.595	7.8*10^−2^	NS
*TLR3*	NM_003265.2	0.825	0.63	-
*TLR4*	NM_138554.2	1.45	0.86	-
*TLR6*	NM_006068.2	1.885	0.17	-
*TLR7*	NM_016562.3	1.78	2.5*10^−3^	0.047
*TLR8*	NM_016610.2 (variant 1)	2.278	9.4*10^−5^	1.8*10^−3^
*TLR8*	NM_138636.3 (variant 2)	2.41	5.3*10^−5^	1*10^−3^
*TLR9*	NM_017442.2	1.453	0.18	-
*TLR10*	NM_030956.2	0.949	0.84	-
*MYD88*	NM_002468.3	1.898	4.1*10^−5^	7.8*10^−4^
*TICAM1*	NM_182919.1	0.87	0.52	-
*TICAM2*	NM_021649	0.6	0.37	-
*LY96*	NM_015364.2	1.28	0.12	-
*TOLLIP*	NM_019009.2	0.964	0.99	-
*TIRAP*	NM_148910.2	0.236	7*10^−2^	NS
*CD14*	NM_001040021.1	2.457	3*10^−2^	NS
*IRAK1*	NM_001569.3	1.26	0.1	-
*IRAK4*	NM_016123.1	1.283	0.3	-

### *TLR8* Protein Expression Study

**Figure 3:**
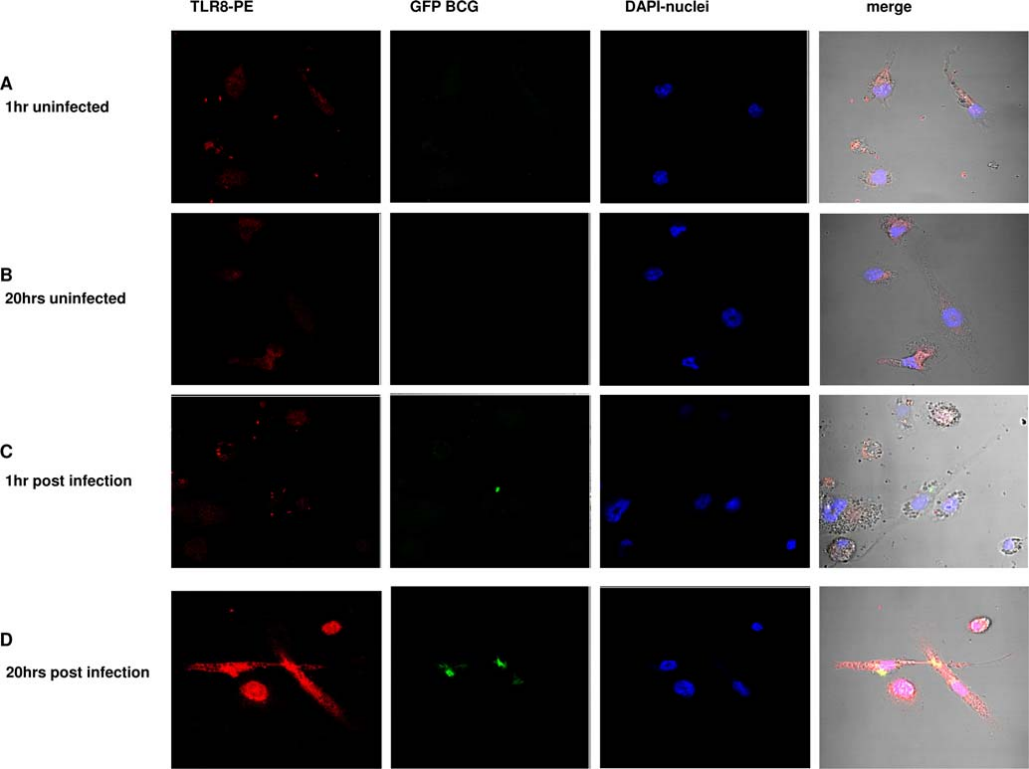
Increased Expression of TLR8 in THP1 cells upon BCG stimulation. THP-1 differentiated macrophages were either uninfected (a,b) or infected with GFP BCG (c,d). Macrophages were harvested for TLR8 expression measured by phycoerytrhin (PE) intensity at 1 hr (c) or 20 hrs (d) post infection and fixed cells were imaged by confocal microscopy.

Expression of TLR8 over time was assessed in human THP1 macrophages after infection by *M. bovis* BCG (Figure 3). Uninfected control cells showed no change of TLR8 protein levels over time (Figure 3A, B). However, infected macrophages showed a marked raise of TLR8 expression at 20 hours post-infection (Figure 3D). It is noteworthy that even cells that didn't take up the whole bacteria displayed an important increase on TLR8 (Figure 3D).

## Discussion

Here we describe a genetic association study aiming to identify polymorphisms within the TLR pathway which confer increased susceptibility to adult pulmonary TB and for the first time report evidence implicating TLR8. Association of the *TLR8* sequence variants with pulmonary TB disease was seen in two independent case-control collections from Indonesia and Russia. Real-time PCR experiments showed the up regulation of TLR8 transcripts in TB patients during acute disease. Protein expression levels of TLR8 were also shown to increase in macrophage cell lines after infection with BCG.

The cloning and characterization of human *TLR7/8/9* revealed significant similarity of their protein sequences [17],[18], defining, together with *TLR3* (GeneID:7098), a new sub-family within the Toll-like receptor genes. In contrast to the other TLRs, their protein products are localized intracellular rather than at the cell surface, mostly in association with the endosomal vacuolar system [19]. Although only TLR9 (NP_059138.1) has been experimentally proven to recognize mycobacterial DNA [20], single-stranded RNA derived from pathogens has been proposed as a likely ligand of TLR7 (NP_057646.1) and TLR8 [21],[22]. The translocation of TLR9 from the endoplasmic reticulum to the lysosome following CpG binding has recently been described [23]. TLR8 and TLR9 are very closely related to each other, raising the possibility that both receptors share a similar mode of activation. *M. tuberculosis* is an intracellular pathogen that resides in characteristic phagosomes, which are not acidic and generally do not mature into phagolysosomes [24],[25]. However, the mycobacterial phagosome interacts with early endosomes, where the bacteria could encounter TLR8.

We found evidence that *TLR8* polymorphisms are associated with susceptibility to pulmonary TB among males. Initially we detected association in the Indonesian population and then observed the same effect in a large independent Russian TB collection, suggesting that this might be a true effect. Nevertheless, our combined evidence (*P* = 6×10^−4^) does not completely exclude association by chance and further studies in statistically powerful sample collections are important. *TLR8* is located on Chromosome X, which suggests that any allele conferring susceptibility to disease may well have a higher impact among males who carry only one copy of the gene. Indeed, the genetic association was more significant in affected males. Hence, inferences about gender-specific effects could possibly be drawn from our findings, where male carriers of rs3764880 allele A showed an increased susceptibility to pulmonary TB. One might expect to find the same association among females homozygous for the same allele, and a tendency towards an altered distribution of affected females homozygous for the minor allele (10%) was indeed observed when compared to female controls (6%) in the Indonesian cohort (Table S2).

In our data the four associated *TLR8* polymorphisms correlate perfectly with each other both in the Indonesian and the Russian samples. Therefore, we were unable to distinguish the variant primarily associated with TB from the polymorphisms associated merely because of linkage disequilibrium. It is also possible that in this study we did not genotype the causal polymorphism. Hence, future experiments should cover all common variation in the associated *TLR8* gene region in order to pinpoint the causal polymorphism. Nevertheless, one of the associated polymorphisms in this study, rs3764880 (Met1Val), is a good functional candidate. Its allele G, associated with protection from TB, abolishes a putative start codon within the alternative transcript variant 2 (Figure 1). Asian populations appear to have an unusually elevated derived allele frequency for this missense variant compared to other ethnic groups [16]. It is remarkable that despite the large differences in allele frequencies between the two populations studied, a genetic association was detected with the same SNPs and in the same direction. Such a significant rise in allele frequency, presumably occurring in Asia, could indicate an important selective advantage or disadvantage for this allele in some environments. Some of the effects of replacing the first methionine of transcript 2 by valine has recently been established [26]. In vitro studies have shown that the G variant affects NF-kappa B activation, as well as response to different TLR8 ligands. Furthermore, the initial amino acids are predicted to act as a signal peptide for this intracellular membrane-bound protein. Therefore, the loss of this sequence could, among other possibilities, affect intracellular trafficking, proper protein folding, or the stability of the mature protein [27],[28]. Further genetic and functional studies of the associated polymorphisms, and other polymorphisms identified by gene resequencing, should add considerably to our understanding of TLR8 function in general, and specifically in response to TB infection.

Two *TLR8* transcript variants have been characterized thus far [17],[18]. Using quantitative RT-PCR we show here that both display significantly upregulated expression in TB patients during the acute phase of their disease, suggesting a functional role for TLR8 during MTB infection. THP1 differentiated macrophages displayed an increase of TLR8 protein levels after infection with *M. bovis* BCG. Interestingly, the rise in expression could be observed even in cells that had not visibly phagocytosed whole bacteria. Although TLR8 ligands remain as yet unidentified, our results are compatible with the hypothesis that a secreted bacterial product might be involved in triggering a TLR8 response, after being taken up by the host cell.

In summary, we report for the first time evidence of associations of *TLR8*, a key gene implicated in the innate immune response, with pulmonary TB in Indonesian and Russian populations. Because it is not expressed in mouse, *TLR8* is among the least studied members of the toll-like receptor family, but our results suggest that it may play a significant role in TB susceptibility, and thus should be the focus of concerted studies in human systems.

## Material and Methods

### Subject Recruitment

#### Indonesia

439 new pulmonary tuberculosis patients above 15 years of age were recruited from an outpatient tuberculosis clinic in central Jakarta (Indonesia) [29]. Diagnosis was based on clinical presentation and chest X-ray examination, confirmed by sputum microscopy positive for mycobacteria [30]. 490 randomly selected control subjects with the same sex and age (+/−10%), were recruited from neighbouring households. First-degree relatives of patients were excluded. Control subjects with signs and symptoms suggesting active tuberculosis or a history of prior anti-TB treatment were also excluded. Self and parental ethnicities were recorded upon recruitment. A Javanese origin characterized three groups - the Jawa, Betawi, and Sunda - and altogether comprised more than 80% of the total sample. The non-Javanese category included individuals born on other Indonesian islands. Subjects were considered of mixed ethnicity when one parent was of Javanese ethnic origin and the other non-Javanese (Table 5).

**Table 5:** Demographic and Clinical Data of the Study Populations.

	Indonesian		Russian	
	TB Patients (N = 375)	Controls (N = 387)	TB Patients (N = 1,837)	Controls (N = 1,779)
Age years (median)	14–75 (28)	15–70 (32)	17–86 (43.8)	16–66 (30)
Gender male(%):female(%)	228(60.8%):147(39.2%)	232(60%):155(40%)	1341(73%):496(27%)	1308(73.5%):471(26.5%)
BCG Scar Present (%)	143 (38%)	168 (43%)	-	-
Self reported ethnicity (%)				
Caucasian	0 (0)	0 (0)	1,837 (100)	1,779 (100)
Javanese	326 (86.9)	314 (81.1)	-	-
Non Javanese	29 (7.7)	21 (5.4)	-	-
Mixed	19 (5.1)	29 (7.5)	-	-
Unknown	1 (0.3)	23 (5.9)	-	-

Prior to recruitment, subjects diagnosed with diabetes mellitus and HIV coinfection, both of which are considered to be major risk factors for tuberculosis development, were not considered. Further tests on recruited subjects were done to confirm absence of diabetes mellitus and HIV coinfection. (Details described elsewhere [31]). Briefly, subjects with levels of fasting blood glucose over 126 mg/dL were considered to have diabetes. HIV testing was performed using dipstick test (Abbott, Determine). Thirty five additional subjects were positive for diabetes.

In order to define a homogeneous phenotype, patients suspected of extra-pulmonary tuberculosis (N = 27) were not considered in the analyses. Controls with suspected tuberculosis after chest X-ray examination (N = 24) or a history of tuberculosis (N = 7) were also excluded.

#### Russia

1,837 cases of pulmonary TB and 1,779 controls were recruited from two Russian cities: St Petersburg and Samara. Clinical data has been described elsewhere [32]. In summary, all TB cases were confirmed by sputum culture of *M. tuberculosis*. Patients with extra-pulmonary TB or HIV-positive were not included in the study. Local blood bank donors with no known history of TB were recruited as controls.

The demographic and clinical data of the Indonesian and Russian cohorts are shown in Table 5. In both groups patients and controls showed a comparable male/female ratio, with males comprising 60% of the subjects in Indonesians and 73% in Russians. Only the Indonesian group had data available on BCG scarring, showing a smaller number of patients with evidence of scarring compared to the control group [38% vs. 43%] (see also ref. 29).

#### Analysis of population stratification

The self-reported ethnicity of each subject and his/her parents was carefully considered in an effort to avoid spurious genetic associations arising from population stratification. In order to detect traces of population stratification in the Indonesian cohort, a large subset of individuals included in this first stage of the study, 330 cases and 368 controls, were genotyped for an independent set of 299 SNPs. One of the SNPs was out of HWE and, thus, excluded from the analysis. These SNPs were chosen to be more than 10 kilobases away from any known gene, to have average minor allele frequencies around 30% and to be in linkage equilibrium with one another. The correction factor was calculated according to the method of Devlin and Roeder [33]. Briefly, an inflation factor was calculated as the median of the chi-square values for all 298 SNPs, divided by 0.675 and then squared. It resulted in a value below 1 (0.82), which indicated that there was no significant population stratification in the Indonesian group (Table S5). Evidence of absence of population stratification in the Russian cohort has been described previously [32].

### DNA and RNA Extraction

Genomic DNA was extracted from whole blood following a protocol described elsewhere [34]. After genotyping, 74 samples were excluded from the Indonesian cohort because of sample duplication and/or familial relationships not originally reported, but identified by RelPair [35].

RNA was successfully extracted using an RNeasy Mini Kit (Qiagen, Germany) from peripheral blood mononuclear cells (PBMCs) of a subset of 23 patients.

Consent forms approved by local Institutional Review Boards of the Medical Faculty of University of Indonesia and the Eijkman Institute for Molecular Biology in Jakarta were signed upon recruitment by all participants. Written informed consents from all Russian subjects as well as permission from ethics committees were obtained [32].

### SNP Genotyping

Selection of SNPs was carried out using an in-house database, GISSNP, which integrates data from public databases (Ensembl, Celera, dbSNP build 123/126) as well as proprietary data. Polymorphisms with the following characteristics were preferentially chosen: putative functional variants resulting in changes in the protein sequence, minor allele frequencies over 5%, average spacing of one SNP every 1 to 2 kilobases. To screen for possible regulatory elements, flanking regions five kilobases upstream and downstream of the gene were also covered.

Design of a custom Oligo Pool Assay (Illumina) was implemented following the manufacturer's specifications. Genotyping was performed with a BeadStation 500G Genotyping System (Illumina). Genotypes were analyzed with Beadstudio software also from Illumina. Ten SNPs were genotyped with a Sequenom primer extension-based protocol described elsewhere [36],[37]. The genotype concordance among the two systems used for genotyping in this study has been reported to be over 99.5% [37]. Assessment of genotypes was done by laboratory personnel without any prior knowledge of the diagnosis of the subjects.

Genotyping of 247 SNPs from 18 candidate genes was performed in the Indonesian cohort. We found that 75 SNPS (30%) of the genotyped polymorphisms were monomorphic in this population (Table S4). Variants with call-rates below 90% (N = 17) were not considered. Six SNPs showed deviations from Hardy-Weinberg equilibrium (HWE) in the control group, and were removed from further analysis (Table S4). Association analyses were applied to the remaining 149 polymorphic SNPs with reliable genotypes (Table S1).

### Genetic Association Statistical Analysis

Hardy-Weinberg equilibrium was calculated in the control group using *HelixTree v4.4.1* (GoldenHelix Inc., Bozeman, MT, United States) and *Exemplar* (Sapio Sciences, LLC, York, PA, United States). Similarly, allelic association analysis was carried out in both software packages. Allelic p-values were calculated by means of a 2×2 chi-square table. A two-sided Fisher Exact test, when counts in any cell fell below five, as well as odds ratios were calculated with *Exemplar*. Allelic analysis of SNPs located on Chromosome X was performed with *Haploview v3.31* [38]. A likelihood ratio test was applied to calculate genotypic associations of SNPs on Chromosome X. Combined p-values were calculated by Fisher's combined probability test which allows pooled information across several tests that share the same null hypothesis [39].

The statistical significance of nominal allelic p-values was assessed by permutation analysis (N = 10,000) with *Haploview v3.31*.

Haplotype blocks and linkage disequilibrium plots were constructed with *Haploview v3.31* using the default algorithm proposed by Gabriel et al [40].

### Taqman Quantitative Reverse Transcription PCR

A subset of 23 patients with active pulmonary TB was selected from a cohort recruited from an outpatient clinic in Jakarta, Indonesia. Blood samples were taken from each of the patients at 2 time points: the active phase of pulmonary TB disease (at the time of admission), and the convalescent phase (6 months after admission and standard anti-tuberculosis multi-drug chemotherapy). Real-time reverse transcription PCR was used to study expression levels of 18 genes (Table S4) on peripheral blood mononuclear cells (PBMCs). Briefly, an aliquot of 10 µl RNA was reverse transcribed to cDNA using the high capacity cDNA kit (Applied biosystems Asia Pte Ltd). The obtained cDNA was diluted 1/5 with water and 10 µl was used for amplification.

### Generation of GFP *M. bovis* BCG

The gfp cDNA cloned into mycobacterial-E.coli shuttle plasmid pMV206 was a gift from Dr. Alain Baulard (Pasteur Institute, France). The plasmid was incorporated into competent BCG cells by electroporation. GFP BCG was observed by the FACS Calibur flow cytometer (Becton Dickinson).

### Differentiation and Infection of THP-1 Cells

The monocytic cell line THP-1(ATCC, Rockville, MD) cells were cultured in RPMI 1640 supplemented with 10%FBS, penicillin (100 U/ml) and streptomycin (100 ug/ml) (Invitrogen). Cells were plated at a density of 2×10^5^/ml in 8-well chamber coverglass (LAB-TEK). Monocytes were allowed to adhere and differentiate into macrophages for 48 hours with 5 nM PMA (Sigma Aldrich) at 37°C in a humidified atmosphere of 5% CO_2_. Differentiated macrophages were infected with GFP BCG at an MOI of 20∶1 and incubated at 37°C, 5% CO_2_. Infected cells at 1 hr post infection were washed twice with RPMI without antibiotics to remove uningested and unadhered bacteria. Cells were then harvested for TLR8 expression as mentioned below. Infected cells at 20 hours post infection were washed to remove uningested and unadhered bacteria at 4 hours post infection to minimize cell death and re-incubated for a further 16 hours. Cells were then fixed with 4% paraformaldehyde (Sigma Aldrich) for 15 minutes at room temperature and cell membrane was disrupted with 1% saponin (Sigma Aldrich) for 20 mins at room temperature. Mouse anti-human TLR8 phycoerytrhin (PE) antibody (Imgenex) was used at 3 ug/ml for 1 hour at room temperature and excess antibody was washed twice with PBS (Sigma Aldrich). To prevent bleaching of the dyes during confocal viewing, anti fade prolong gold with DAPI (Invitrogen) was added to slides so that it formed a protective layer over the cells. Slides were stored at 4°C in the dark until confocal viewing.

### Confocal Image Acquisition

The LSM 510 scanhead of the confocal laser-scanning microscope system (Zeiss 5 duo, Germany) was used to detect intracellular fluochrome. Cells were scanned by triple excitation for PE (red), GFP (green) and DAPI (blue) fluorescence. A 63× oil objective with numerical aperture of 1.4 was used and images were captured.

## Supporting Information

Table S1 Location, Allele, Genotype Frequencies, and Allelic p-values of 149 Analysed SNPs.
(0.06 MB XLS)

Table S2 Genotype Distribution of TLR8 Polymorphisms among Indonesian TB Patients and Controls in All and Females.
(0.02 MB XLS)

Table S3 Haplotype Analysis and Distribution of TLR8 Polymorphisms among Male TB Patients and Controls from Both Cohorts.
(0.02 MB XLS)

Table S4 Candidate Genes and SNPs Analyzed.
(0.02 MB XLS)

Table S5 Allele Frequencies of 298 SNPs Tested for Population Stratification in the Indonesian Cohort.
(0.07 MB XLS)
